# Genomic Islands in Mycoplasmas

**DOI:** 10.3390/genes11080836

**Published:** 2020-07-22

**Authors:** Christine Citti, Eric Baranowski, Emilie Dordet-Frisoni, Marion Faucher, Laurent-Xavier Nouvel

**Affiliations:** Interactions Hôtes-Agents Pathogènes (IHAP), Université de Toulouse, INRAE, ENVT, 31300 Toulouse, France; emilie.dordet-frisoni@envt.fr (E.D.-F.); marion.faucher@ipbs.fr (M.F.); xavier.nouvel@envt.fr (L.-X.N.)

**Keywords:** genomic island, mobile elements, phages, horizontal gene transfer, *Mollicutes*, mycoplasmas, evolution, genome

## Abstract

Bacteria of the *Mycoplasma* genus are characterized by the lack of a cell-wall, the use of UGA as tryptophan codon instead of a universal stop, and their simplified metabolic pathways. Most of these features are due to the small-size and limited-content of their genomes (580–1840 Kbp; 482–2050 CDS). Yet, the *Mycoplasma* genus encompasses over 200 species living in close contact with a wide range of animal hosts and man. These include pathogens, pathobionts, or commensals that have retained the full capacity to synthesize DNA, RNA, and all proteins required to sustain a parasitic life-style, with most being able to grow under laboratory conditions without host cells. Over the last 10 years, comparative genome analyses of multiple species and strains unveiled some of the dynamics of mycoplasma genomes. This review summarizes our current knowledge of genomic islands (GIs) found in mycoplasmas, with a focus on pathogenicity islands, integrative and conjugative elements (ICEs), and prophages. Here, we discuss how GIs contribute to the dynamics of mycoplasma genomes and how they participate in the evolution of these minimal organisms.

## 1. Common and Specific Features of Mycoplasma Genomes

Members of the *Mycoplasma* genus belong to the *Mollicutes*, a large class of bacteria characterized by the lack of a cell-wall. Mycoplasmas also distinguish themselves by their small, pleomorphic cell, the use of UGA as tryptophan codon instead of the universal stop, and their simplified metabolic pathways. These features are all directly linked to the small-size and limited-content of their genomes (580–1840 Kbp; 482–2050 CDS) that have nevertheless retained the full capacity to synthesize DNA, RNA, and all the proteins required to sustain a parasitic life-style. Indeed, the *Mycoplasma* genus encompasses over 200 species, all colonizing and living in close contact with their hosts, including man and a wide range of animals. Still, most species are able to grow in rich media under laboratory conditions, making some of these bacteria the smallest and simplest known organisms capable of autonomous replication [1].

Due to their general simplicity, mycoplasmas were first considered as primitive organisms from which more complex bacteria emerged during evolution. This assumption was abandoned in the 1980s, following Carl Woese’s phylogenetic analyses based on 16S rRNA—*Mollicutes* derived from a common ancestor to Gram-positive bacteria with a low GC content [2]. Phylogenomics more recently supported these data and confirmed the monophyletic origin of *Mollicutes* within the *Firmicutes* [3]. *Mollicutes* were further divided into 4 main related phylogenetic clades, three of which (Hominis, Pneumoniae, and Spiroplasma) contain members of the *Mycoplasma* genus, with some being positioned on some of the longest branches of the universal phylogenetic tree [3]. This finding points towards mycoplasmas as some of the fastest evolving bacteria [4]. 

The small-size of the mycoplasma genome (under 1 Mbp) is well suited for whole genome sequencing (WSG) and the circular, 580-Kbp chromosome of the human pathogen, *Mycoplasma genitalium* was one of the first bacterial genomes to be fully decrypted [5]. Currently, genome sequences in public databases are available for over 60% of the known mycoplasma species and for more than 280 strains, of which approximately half are provided as a single circularized chromosome. These numbers are increasing rapidly but the already existing data offer a valuable source for mining the pan mycoplasma genome. Indeed, comparative genome analyses combined with saturation transposon mutagenesis have been conducted for more than 20 years with the aim (i) of exploiting mycoplasmas for the study of the minimal cell concept and identifying the set of essential genes required to sustain life and (ii) for identifying virulence genes in pathogenic species. Depending on the species considered and the method, the estimated number of essential genes varied from 256 to 422 [6,7,8]. An important part of the remaining gene pool turned out to encode hypothetical proteins with little homology to virulence factors identified in other, more typical bacteria.

Since the 1990s, studies combining comparative genomics to classical microbiology approaches showed that several mycoplasma species, most specifically those infecting ruminants, exchanged significant parts of their genomes during evolution and still retained the ability to conjugate [9,10,11]. Horizontal gene transfer (HGT) is one of the main drivers of microbial innovation and is responsible for the exchange of large gene clusters, known as genomic islands (GIs) among bacteria. This review summarizes our current knowledge regarding GIs found in mycoplasmas and discusses their particular features with regards to those found in bacteria with larger genomes. Several types of mobile genetic elements fall into the broad definition of GIs and the focus here is on pathogenicity islands, integrative and conjugative elements (ICEs), and prophages (integrated phages).

## 2. Genomic Islands and the Mycoplasma Flexible Gene Pool

Comprehensive analyses of bacterial genomes revealed the presence of GIs as part of the flexible gene pool [12]. Present in pathogenic or nonpathogenic bacteria, these are usually gene clusters spanning several Kbp that have potentially been acquired by HGT, as reflected by their GC content or codon usages, which might differ from the rest of the core genome. GIs often encode virulence or adaptive traits and are frequently associated with tRNA or integrase genes located at one end of the island. Most GIs derived from mobile genetic elements have lost their mobility genes through evolution but some have retained the ability to disseminate from cell-to-cell via conjugation or transduction. These are respectively known as ICEs and prophages. 

Until the 1990s, the occurrence of GIs and HGT in mycoplasma was not considered, due to the small size of their genome and the prevailing evolutionary scenario that was only based on successive gene losses [13]. Yet, as more strains from a single mycoplasma species were being sequenced, important discoveries were made that challenged this paradigm [4,14,15]. One relates to the occurrence of ICEs in several mycoplasma species (see below), with their distribution being variable among strains of the same species. The second was based on comparative genomics across strains and species, revealing that HGT has shaped the genome of ruminant mycoplasmas raising the prospect that in addition to ICEs, mycoplasma genomes might harbor other GIs. Current tools for GI prediction are unfortunately often not reliable when using mycoplasma genomes. These prediction tools, which are based on sequence composition or comparative genomics, might be less suited (i) to the low GC-content and biased genetic code of the mycoplasma genome, and (ii) to the low homology existing between mycoplasmal genes and classical virulence factors or mobile genetic elements. 

**Figure 1:**
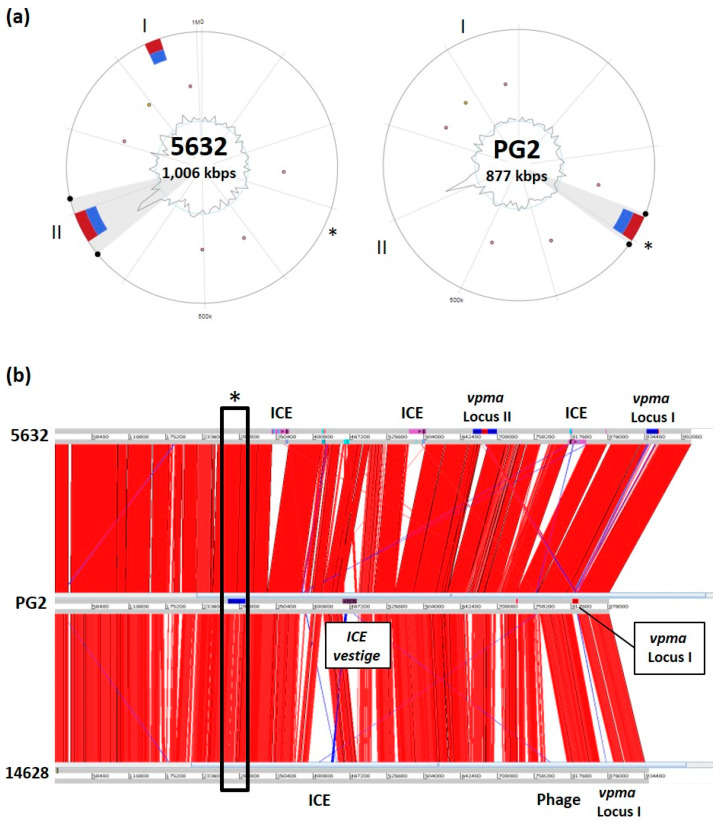
Genomic island (GIs) predictions and locations in three sequenced *M. agalactiae* strains. (**a**) GI predictions using Island Viewer 4 and the two circular genomes of *M. agalactiae* strain 5632 (left) and type strain PG2^T^ (right). The circle represents a single chromosome, with the outermost red bars indicating locations of all predicted GIs by integrating the four detection methods included in IslandViewer 4. Within the circle, GIs predictions by the software IslandPath-DIMOB are shown as blue. SIGI-HMM, IslandPick, and Islander did not give any result. Homologs to microbial resistance genes and pathogen-associated genes are indicated as circular glyphs inside the circles. (**b**) Genome comparison using the Artemis Comparison Tool and the *M. agalactiae* genome from 3 strains, 5632 (NCBI RefSeq NC_013948.1), PG2 (NCBI RefSeq NC_009497.1), and 14628 (WGS SPQY01000001: SPQY01000015). The *vpma* loci are labeled I and II and are detailed in Figure 2; the asterisk designates the locus detected in PG2^T^ by Island Viewer 4. *M. agalactiae* ICEs present in 5632 and 14628 are indicated as well as the position of an ICE vestige in PG2. A prophage identified only in 14628 is shown, which was not detected by Island Viewer 4 or other dedicated prediction tools. Of note, panel (a) was generated using genomes annotated with the NCBI Prokaryotic Genome Annotation Pipeline (PGAP) (NC_009497.1 for PG2 and NC_013948.1 for 5632), when expert annotation was used, one ICE was detected in strain 5632, in addition to the *vpma* loci and a single locus in PG2, which was not detected before (CU179680.1 for PG2; FP671138.1 for 5632).

**Figure 2:**
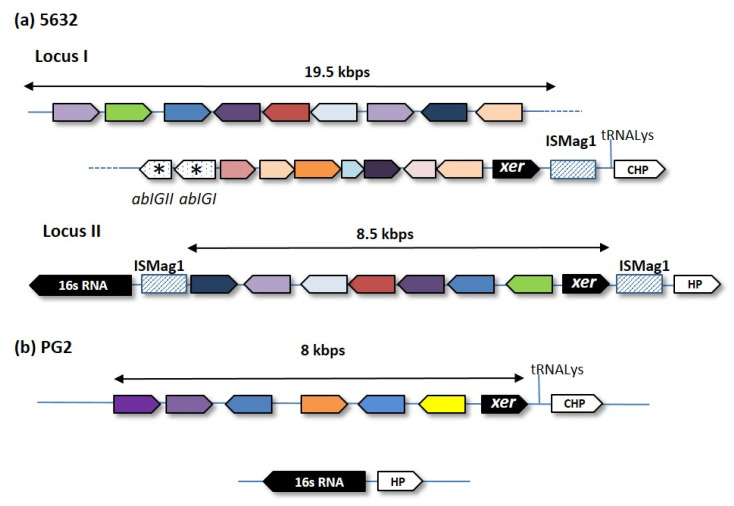
Comparison of *M. agalactiae vpma* loci of strain 5632 (**a**) with that of the PG2^T^ type strain (**b**). In PG2^T^, the counterpart of 5632 locus-II is deprived of *vpma* genes. Large filled arrows represent Vpma CDSs, with each color representing individual Vpmas whose genes might occur in the two loci. The two non-Vpma-related CDSs (*abiG*I and *abiG*I) only found in 5632 are indicated by an asterisk. IS*Mag1* elements are indicated by hatched boxes. Schematics were approximately drawn to scale. HP—hypothetical protein; CHP—conserved hypothetical protein. Locus I of 5632 was split in two parts to fit into a portrait format but were indeed collinear, as indicated by the dotted lines. *xer*: genes encoding the tyrosine recombinase [20].

Current GI predictive tools can however provide hints for further expert analyses. For instance, Island Viewer 4 detected two loci in the *M. agalactiae* strain 5632 [16] (Figure 1), a small ruminant pathogen for which the circular genome is available [17]. Close examination revealed that these loci carry a cluster of related genes, each encoding for surface proteins of the Vpma family [18]. The *vpma* gene organization of strain 5632 is depicted in Figure 2 and showed that these loci harbor several features characteristic of GIs, as described for other bacteria. More specifically, the two loci span several Kbp (19 Kbp for 5632-locus I), contain a recombinase gene at one end, are in close proximity of mobile insertion sequence (IS) elements, and vary in their *vpma* content among strains [19]. In addition, the largest cluster, locus I, is closed to a tRNA_Lys_ gene and harbors two unrelated genes, *abi*GI and *abi*GII, whose products present similarities to the abortive infection system AbiG found in more classical bacteria. 

Discovered in the 90s, the *M. agalactiae vpma* loci were shown to encode phase-variable surface proteins that are key in allowing host-colonization and immune-escape [21,22]. These GIs could match the definition of a pathogenicity island (PAI), a GI involved in virulence. The *M. agalactiae* PG2 type strain carries a single *vpma* locus whose location corresponds to locus I of 5632 but was not detected by the prediction tool. Rather, Island Viewer 4 points towards a region encompassing 20 genes, distant from the *vpma* cluster, which is present in both 5632 and PG2 but was only detected in PG2 (Figure 2). Whether this also represents a GIs was less clear but close examination of this locus revealed the presence of coding sequences (CDSs) encoding several surface proteins, including the P40, which was involved in adhesion to host cell [23] and that of a CDS with homology to RumC recombinases. 

The *M. agalactiae vpma* family had a counterpart in its close relative *M. bovis* [24], a pathogen of cattle, and is, to our knowledge, the only pathogenicity island (PAI) described as such in mycoplasmas. Several gene clusters, some encoding virulence factors, have undergone HGT among mycoplasmas [9], but lacked some features found in classical GIs. 

## 3. Mycoplasma Integrative Conjugative Elements

Mycoplasma ICEs (MICEs) are self-transmissible elements that play a crucial role in the unconventional chromosomal transfer described in *M. agalactiae*; their contribution toward HGT in mycoplasma was reviewed in 2018 [14], and here the focus is on their genetic organization and specific features.

MICEs were initially discovered in *M. fermentans* and *M. agalactiae* that were respectively isolated from human and ruminants [25,26]. In these two mycoplasma species, sequences with localized homologies to two gene-encoding proteins with DNA mobility-related functions in other bacteria [27], namely *traG* and *traE*, were detected in a chromosomal region having a GC-content slightly different than flanking genomic sequences [25]. Thus far, data mining of the mycoplasma genomes retrieved MICEs in 14 different mycoplasma species isolated from ruminants, swine, or human that belonged to two phylogenetic clades, namely Hominis and Spiroplasma. Recurrent attempts to automatically detect MICEs in sequenced mycoplasma genomes failed when using dedicated software (i.e., using ICEfinder tool of ICEberg 2.0 [28]), leaving manual search based on sequence similarities of conserved MICE genes the only reliable approach. While this situation reflects the little overall homology existing between MICE and ICE from other, more classical bacteria (see below), it is expected that the growing number of MICE sequences in databases should improve their future detection.

**Figure 3:**
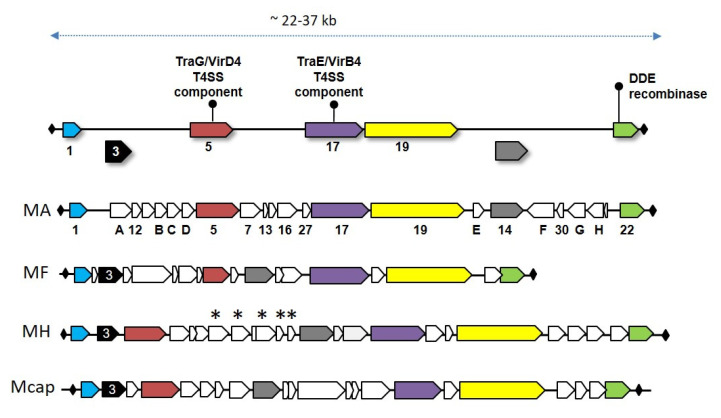
Overall gene organization of Mycoplasma Integrative and Conjugative Element (MICE). MICEs are large GIs whose sizes range from 22–37 kbp and display a set of highly conserved genes across MICEs. These are represented as colored filled arrows with some, less conserved, being represented below the line. Inverted repeats that flank the MICE and are juxtaposed in the circular form are represented by diamonds. Direct repeats (not shown here) are generated upon MICE random integration in the host chromosome. For illustration, representative MICEs of the *M. agalactiae* strain 5632 (MA), *M. fermentans* strain M64 (MF), *M. hominis* strain 4788 (MH), and *M. mycoides* subsp. *capri* strain GM12^T^ (Mcap) are shown. Asterisks represent MICE genes which are specific to *M. hominis* ICEs.

MICEs are large modular chromosomal regions of 22 to 37 Kbp that encode for about 20 structural genes flanked by two inverted repeats (IR), which are juxtaposed in the free circular form (Figure 3 and Table 1). One of their hallmarks is the occurrence at their 3′ end of a structural gene, CDS22, which encodes a DDE recombinase belonging to a family of prokaryotic DDE transposases, themselves related to eukaryotic Mutator-like transposases [29]. In 2014, Guerillot et al. systematically searched for genetic elements carrying DDE transposases and showed that they were associated with large integrative elements in streptococci (Tn*GBS*) and with MICEs, which clustered in one large family designated p-MULT 5 [29]. Tn*GBS* preferentially insert upstream streptococci promoters and this particular insertion was associated with a specific signature not found in CDS22 [30,31]. Indeed, MICEs belong to the few conjugative transposons which integrate at random in their host chromosome, with the excision-integration process being driven by the DDE recombinase encoded by CDS22. This finding was supported by several direct and indirect evidences, including the monitoring of the integration of a mini-artificial ICE construct in *M. agalactiae*, genome sequencing of MICE flanking sequences and comparative genomics of MICEs in different species [30,31].

As identified by transposon mutagenesis or BLAST searches, a large portion of MICE genes are involved in the horizontal self-dissemination of the element [46]. MICEs also carry several hypothetical genes with no predicted function or no homology, with some being specific to a single mycoplasma species. For instance, *M. hominis* ICEs all possess a 4.0–5.1 Kbp cluster of five to six CDSs (Figure 3) with no homolog in other MICEs and whose synteny slightly differs among strains [42]. Except for one, all of these were predicted to share structural similarities to DNA interacting/modifying proteins, with some sharing common structural similarities with transcription activator-like (TAL) effectors. In symbiotic bacteria, TAL effectors are involved in polynucleotide recognition and signal transduction. Interestingly, *M. hominis* colonizes the human urogenital tract, where it might occur as an endosymbiont of *Trichomonas vaginalis*. Whether TAL effectors encoded by *M. hominis* ICEs contribute to the interaction of the mycoplasma with *T. vaginalis* by impacting gene expression is an interesting hypothesis that remains to be addressed [42]. 

Entire, functional MICEs often occur in multiple copies in a single mycoplasma genome, along with MICE vestiges, as a result of MICE erosion (Table 1) [17]. This raised the question of the cost imposed by these large, multi-copy elements on the fitness of the small mycoplasma genome. We recently showed that the *M. agalactiae* strain 5632 that harbors 3 MICEs copies is more fit under laboratory conditions than the PG2 strain that only has a single MICE vestige (Figure 1) [47]. Following mating experiments, the transfer of one MICE from 5632 into the PG2 genome resulted in fitness loss. This finding suggests that strain 5632 is able to counterbalance the fitness cost of MICEs, while PG2 is not. Comparative genome analyses showed that the two strains encode syntenic genomes but differ in several SNPs and the size of their variable gene repertoires, such as the *vpma* (see above and Figure 2) [17]. Yet, the factors responsible for 5632 adaptation to MICEs cost remain to be explored. Of note, MICE deletion in *M. mycoides* subsp. *capri* strain GM12^T^ did not affect the in vitro growth [48].

As already mentioned, all MICEs described above belong to a new family of self-transmissible elements that have so far not been found outside of *Mollicutes* and thus appear to be specific to this class. Yet, MICEs are not the only large mobile genetic elements (MGE) circulating in mycoplasmas. Indeed, an excision-competent composite transposon was identified in *M. hominis*, which derived from a larger uncharacterized transposon of *Streptococcus agalactiae* and carried the *tet*(M) determinant, along with Tn*916* sequences [43]. Tn*916* and Tn*916*-like conjugative transposons encode the *tet*(M) determinant that confers resistance to tetracycline. These were found in several bacterial genera but have seldom been detected in *Mollicutes*. Thus, the finding of this composite transposon in *M. hominis* indicates that ICEs from two different origins circulate in this mycoplasma species and that tetracycline resistance might disseminate via HGT. 

## 4. Mycoplasma Viruses and Prophages

The identification of prophage sequences in a growing number of mycoplasma species indicates that GIs in *Mollicutes* are not limited to MICEs and raises questions about the influence of these particular elements on mycoplasma genomic evolution and environmental adaptation. 

The first direct evidence of a viral attack in *Mollicutes* was provided by the isolation of a plaque producing phage in *Acholeplasma laidlawii* [49]. Since then, several groups of viruses were documented in the genus *Acholeplasma*, and this repertoire quickly extended to phylogenetically distant genera, including *Spiroplasma* and *Mycoplasma* [50,51,52,53]. Acholeplasma (*Acholeplasmataceae*) and Spiroplasma (*Spiroplasmataceae*) viruses were extensively characterized. These viruses were classified into several groups with specific morphological and genomic features [51,52]. Remarkably, the L2 group belonged to the *Plasmaviridae*, a unique family of enveloped viruses only represented by the Acholeplasma virus L2. The genome of this virus was encoded by a circular double-stranded DNA molecule that could be found integrated into the host chromosome at tRNA genes. The other groups belonged to *Inoviridae* and *Podoviridae*, two major viral families that unlike *Plasmaviridae* encompass an important number of Gram-positive and Gram-negative targeting bacteriophages. Yet, many of these viruses still had an uncertain taxonomic classification.

With more than 200 species, members of the *Mycoplasma* genus are associated with a considerable number of chronic infections in humans and a broad range of animal hosts. In contrast to the many Acholeplasma and Spiroplasma viruses identified, only four Mycoplasma viruses (MV) were successfully isolated so far (Table 2). MVBr1 was isolated from *M. bovirhinis*, a commensal species frequently found associated with the upper respiratory tract in cattle [54,55]. With its isometric head and long tail, MVBr1 has the typical bacteriophage structure and was classified in the family *Myoviridae*. This bacteriophage contains a linear dsDNA molecule of about 11.7 Kbp. Three other viruses were isolated from pathogenic mycoplasma species (Table 2), but their physicochemical properties were not fully characterized and thus had no taxonomic status.

As mycoplasma genome sequences became available in public databases, a growing number of genomic elements were identified as prophage genomes or phage-like sequences [56,60,61,63,64,66,70]. Consistent with our previous observations with MICEs (see above), most existing computational tools dedicated to identifying prophage sequences in bacterial genomes are poorly efficient in distinguishing viral patterns from mycoplasma sequences [71]. Yet, as suggested below by BLASTP analyses using available mycoplasma viral sequences (Table 2), the number of detected prophages in published mycoplasma sequences is most likely underestimated. Thus, the dogmatic view suggesting that mycoplasmas were subjected to the elimination of prophage genomes during their evolution might have to be revisited [72]. 

**Figure 4:**
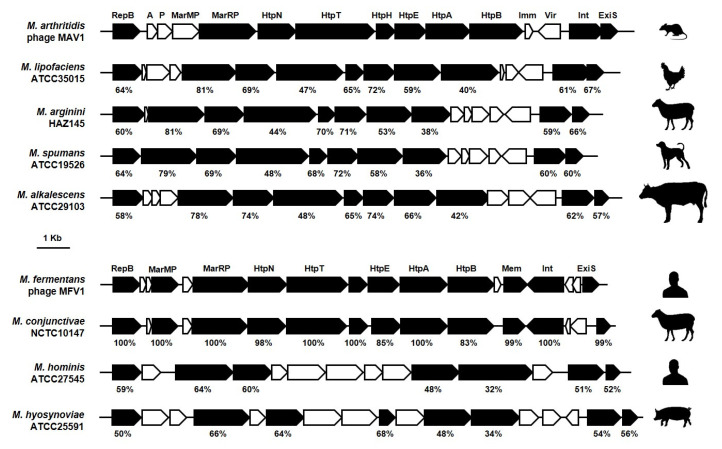
Comparison of φMAV1 and φMFV1 genomic regions with prophage sequences identified in mycoplasma species of the Hominis phylogenetic group. φMAV1-like and φMFV1-like sequences were identified by BLASTP analyses using CDSs highlighted in black and a sequence database consisting of non-redundant protein sequences restricted to *Mollicutes* (taxid:31969). No similarity was identified outside of the Hominis phylogenetic group. CDS products in φMAV1 and φMFV1 are indicated. For each CDS, the percentage of global similarity with φMAV1 or φMFV1 is indicated. This value was determined by using the EMBOSS Needle alignment tool. Animal icons are used to illustrate the host tropism of each strain. These illustrations are limited to a single prophage sequence per chromosome, and a single strain per species. The overall organization φMAV1 is highly conserved among φMAV1-like sequences, which mainly differ at putative repressor (*imm*) and virulence (*vir*) genes and CDSs located at close proximity. A remarkable feature of φMFV1 genomic region is its proximity with the animal *Mycoplasma* species *M. conjunctivae*.

Based on their overall genetic organization and sequence similarity, mycoplasma prophage genomes can be divided into two groups (see below). The first group comprises prophages with a small genome in size (genome size ca. 16 Kbp) represented by *M. arthritidis* φMAV1 and *M. fermentans* φMFV1 that have a similar genetic organization and a high degree of synteny [53,63]. Both genomes exhibit characteristic features of mobile genetic elements, including a compact organization, an almost unidirectional CDS orientation, and the occurrence of genes involved in their integration and excision from the host chromosome [53,63]. BLASTP analyses revealed that 10 of the 18 CDSs identified in φMFV1 exhibited some similarity with their counterparts in φMAV1 [63]. Here, database searches of available genomes revealed prophage sequences that closely resemble φMAV1 or φMFV1 and were broadly distributed among human and animal mycoplasma species (Figure 4) These included the *M. hominis* φMHoV1 [64], as well as several *M. hyosynoviae*-derived phages [66]. In this highly conserved genomic framework are several regions of higher heterogeneity (Figure 4). Among those are the φMFV1 *mem* and the φMAV1 *vir* products, each encoding a unique membrane-anchored surface protein [58,59,63,73]. Mem displays features of coiled-coil proteins and occurs predominantly as an integral, membrane-associated product anchored by its N-terminal transmembrane domain. The expression of this phage product can vary among clonal populations of *M. fermentans*, without affecting the viability of mycoplasma cells [63]. The φMAV1-specific *vir* gene was found to encode a lipoprotein expressed at the surface of *M. arthritidis* infected cells and to confer resistance to phage superinfection [73]. Interestingly, φMAV1 was reported to be required for arthritogenesis in rodents, and the phage-encoded Vir lipoprotein was identified as a putative virulence factor [53,59,74]. However, attempts to confirm this hypothesis were unsuccessful, suggesting a complex interaction between φMAV1 and *M. arthritidis* in the infected host [53,73,75]. The biological impact of phage-encoded surface proteins is still unclear, but these studies illustrate the possible role of prophage-associated genes in the acquisition of new heritable phenotypic traits in mycoplasmas. 

**Figure 5:**
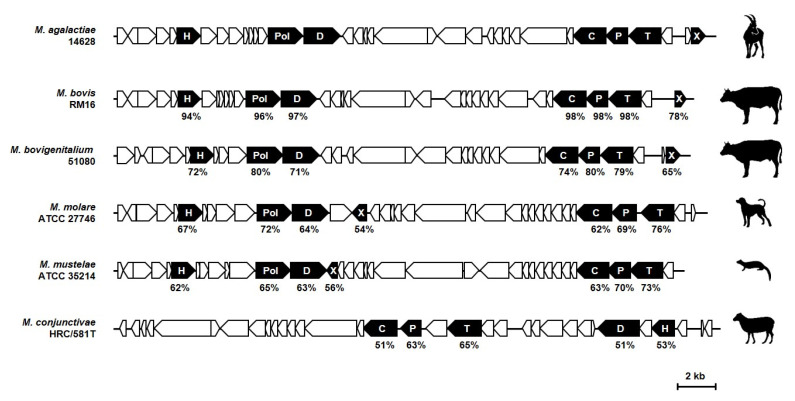
Genome map of *M. agalactiae* φMAgV1 from strain 14628 and comparison with MAgV1-like sequences identified in *Mycoplasma* species of the Hominis phylogenetic group. The locations, sizes, and orientations of the CDSs are indicated by arrows. MAgV1-like sequences were identified by BLASTP analyses using φMAgV1 CDSs highlighted in black and a sequence database consisting of non-redundant protein sequences restricted to *Mollicutes* (taxid:31969). No similarity was identified outside of the Hominis phylogenetic group. The letter code in black arrows refers to CDS products: H, helicase; Pol, DNA polymerase; D, DNA primase; C, prohead protein; P, portal; T, terminase; X, Xer. For each CDS, the percentage of the global similarity with φMAgV1 is indicated. This value was determined by using the EMBOSS Needle alignment tool. Animal icons are used to illustrate the host tropism of each strain. The overall organization of φMAgV1 is conserved in *M. bovis* RM16 and *M. bovigenitalium* 51080. A particular feature of MAgV1-like sequences in *M. molare* ATCC 27746 and *M. mustelae* ATCC 35214 is the central position of the recombinase gene (*xer*). The genome of *M. conjunctivae* HRC/581T is characterized by an important reorganization of the MAgV1-like sequence and the absence of DNA polymerase and recombinase genes.

The second category of mycoplasma prophages displays a larger genome (size ca. 34 Kb), which were identified in several ruminant mycoplasma species, including *M. agalactiae*, *M. conjunctivae*, and *M. bovigenitalium* [41,56]. Their genetic architecture is an assemblage of several regions, each characterized by a similar number of CDSs sharing the same orientation. In contrast to φMAV1 and φMFV1, sequences with a high degree of similarity are limited to several common phage products (Figure 5). BLASTP analyses using several of these typical phage products, such as the prohead protein, portal, and terminase of the *M. agalactiae* prophage [56], not only confirmed the occurrence of a similar prophage region in *M. conjunctivae* and *M. bovigenitalium* but also identified new host species, including the ruminant pathogen *M. bovis*, as well as several mycoplasmas isolated from the respiratory tract of dogs and minks (Figure 5). The *M. agalactiae* prophage, hereafter designated as φMAgV1, was reported in the genome of an atypical strain associated with a mortality episode of Alpine ibexes in France [56].

**Figure 6:**
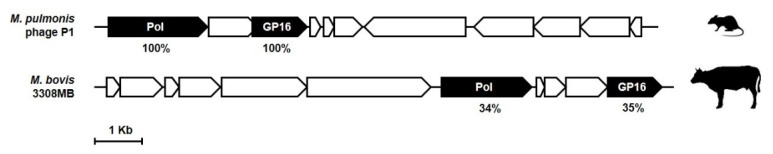
Genomic organization of *M. pulmonis* phage P1 and putative fingerprint of a P1-like phage attack in *M. bovis* 3308MB. Black arrows indicate CDSs in phage P1 displaying some similarity with sequences of the 3308MB draft genome. The percentage of global similarity was determined by using the EMBOSS Needle alignment tool. The letter code in black arrows refers to the CDS products. Pol, DNA polymerase GP16, DNA encapsidation protein. Animal icons are used to illustrate the host tropism of *M. pulmonis* and *M. bovis*.

Apart from these two prophage groups, an important number of phage-like protein sequences were recently documented in a 54 Kbp chromosomal region of *M. bovirhinis* strain HAZ141_2 suggesting the possible occurrence of a unique prophage in this ruminant mycoplasma species [60,61]. Proteins with significant similarity to known phage proteins were also detected in the genome of several human urogenital *Mycoplasma* species [70]. The origin of these sequences, detected by using the PHASTER web server [76], is largely unknown, but their clustering within one or two chromosomal regions raises the possibility of a common origin. While the extraordinary diversity of prophage-like sequences in mycoplasmas has only started to emerge, BLASTP analysis might also be useful to identify possible footprints of viral attacks in mycoplasmas. This was observed in the draft genome sequence of *M. bovis* strain 3308MB, which was found to harbor sequences with some similarity to the *M. pulmonis* virus P1 (Figure 6). Interestingly, these sequences were found within a single contig of the draft genome with an overall organization similar to *M. pulmonis* virus P1 and no sequence homology with *Mollicutes*. The viral origin of this single contig sequence remains to be confirmed, but it might indicate that the mycoplasma isolate used for genome sequencing was facing a viral attack. 

Finally, the isolation of plaque-forming viral particles for φMAV1 [59], the detection of extrachromosomal forms of φMFV1 and φMAgV1 [56,63], together with the identification of multiple chromosomal integration sites [63], demonstrated the remarkable mobility of these prophage genomes and their contribution to genotypic variation.

## 5. Distribution of MICE and Prophages in *Mycoplasma* Species

The prevalence of MICE among strains depends on the species:98% of *M. bovis* strains tested so far carry MICE sequences of the minimal backbone, that is, the set of MICE genes common to all (Figure 3), for only 34% in the closely related *M. agalactiae* species or 45% in *M. hominis* clinical isolates [30]. Notably, some *Mycoplasma* species with several strains sequenced seemed to be deprived of MICE. This is the cases of *M. genitalium*, *M. pneumoniae*, or *M. gallisepticum*, which have different genome size and colonize different hosts—the two first ones colonize the human genital and respiratory tracts, respectively, and have genomes of 580 and 820 Kbp; the third one is a respiratory pathogen of birds and has a genome size of about 990 Kbp, similar to that of *M. agalactiae* 5632 (ca. 1000 Kbp). These 3 species belong to the phylogenetic Pneumoniae clade and MICEs were only identified so far in mycoplasmas of the Spiroplasma and Hominis clades. Whether there is a link between the occurrence of MICEs in a given species and its phylogenetic clustering, remains to be explored, but there are evidences of MICE dissemination across the Spiroplasma and Hominis clades [30].

As for MICEs, the occurrence of mycoplasma prophages varied within species. For instance, the *M. agalactiae* φMAgV1 was detected in most but not all ibex isolates and was absent from most ovine strains [56]. This phage was inserted in a region that might have undergone horizontal gene transfer with members of the mycoides cluster, a remote group of ruminant species, suggesting that this prophage might be directly or indirectly associated with genome dynamics. Another example of the intricate link between prophage and genome dynamics is the occurrence of several φMFV1 regions in one copy of *M. fermentans* MICE in strain PG18 [26,63]. This illustrates the possibility of gene exchange between these regions and suggests that MICE might contribute to the horizontal dissemination of prophage genes. 

Several restriction-modification, bacteriophage abortive infection mechanisms (Abi), and CRISPR systems were identified in mycoplasmas. These are known to restrict HGT, and in mycoplasmas, their repertoire varies within species [77,78,79]. Whether the strain-specificity of these systems correlates with the distribution of MICEs and prophages within a single species is yet to be fully addressed but there is so far no apparent correlation. 

## 6. Conclusions

Since the 1990s, the main scenario proposed to explain *Mollicutes*’ evolution was based on gene losses. The discovery of MICEs had two main impacts: toa break away from the general idea that small mycoplasma genomes were deprived of complex mobile genetic elements and to stimulate research on mycoplasma horizontal gene transfers. Based on comparative genomics and mating experiments, it is now clear that several *Mycoplasma* species retained a form of sexual competence and that their evolution was also driven by gene gain. The mechanism underlying HGT in ruminant *Mycoplasma* species hasstarted to emerge and may challenge the definition of GIs in these bacteria. Indeed, in these organisms *in silico* and experimental data points towards the entire genome being mobile, with large genomic chromosomic regions being exchanged. 

This review showed that several mycoplasma genomes are populated with MICEs and prophages, and most likely, also contain pathogenicity islands. The identification of these particular GIs might be difficult in certain species because of the unconventional mechanism of HGT that shuffle their genomes. In addition, the specificity of the mycoplasma genetic code most likely limits the acquisition of GIs from donor bacteria outside of *Mollicutes*.

This review also points towards the heterogeneity of the species pan-genome, underlying the need for exploring multiple strains even in bacteria with small genomes.

Overall, a new picture has started to emerge since the last 10 years in which mycoplasma genomes are more dynamic than first thought.

## Figures and Tables

**Table 1:** Mycoplasma integrative conjugative elements (MICE).

Phylogenic Group	Species	Hosts ^1^	Strain	ICE Occurrence	ICE Designation	Copy Number	Size (Kbp)	CDS Number	Reference
Hominis	*M. conjunctivae*	Small R	HRC/581 ^T^	+	ICECJ	2	28.5	23	[32]
	*M. hyopneumoniae*	Swine	7448	+	ICEH	1	22.3	14	[33]
			168	+	ICEH	1	23.4	19	[34]
			232	+	ICEH	1	22.5	19	[35]
	*M. fermentans*	Human	PG18 ^T^	+	ICEF	4	23.2	22	[26]
			JER	+	ICEF	2	24.4	23	[36]
			M64	+	ICEF	7	22.7–26.0	22–26	[37]
	*M. agalactiae*	Small R	PG2 ^T^	vestige	vestige	1	nd	nd	[9]
			5632	+	ICEA	3	27.2	23	[25]
		Wild R	14628	+	ICEA	1	27.2	23	[30]
	*M. bovis*	Cattle	PG45 ^T^	+	ICEB	1	37.1	22	[38]
			Hubei	vestige	vestige	un ^4^	nd	nd	[39]
			HB0801	vestige	vestige	un	nd	nd	[40]
	*M. auris*	Small R	15026	D ^2^	nd ^3^	un	nd	nd	[31]
	*M. bovigenitalium*	Cattle	51080	D	nd	un	nd	nd	[41]
	*M. alkalescens*	Cattle	14918	D	nd	un	nd	nd	[41]
	*M. hominis*	Human	4788	+	ICEHo	1	29.1	25	[42]
			4235	+	ICEHo	2	30.5	25	[42]
			35	+	ICEHo	2	29.1; 30.3	25	[42]
			Sprott	+	TetM mosaic transposon	1	25.2	11	[43]
Spiroplasma	*M. mycoides* subsp. *capri*	Small R	GM12 ^T^	+	ICEM	1	29.6	21	[30]
			95010	+	ICEMx2	2	30.0	21	[44]
	*M. capricolum* subsp. *capricolum*	Small R	CK ^T^	+	ICEC	1	23.8	17	GenBank CP000123.1
	*M. putrefaciens*	Small R	9231	D	nd	un	nd	nd	[45]
	*M. yeatsii*	Small R	13926	D	nd	un	nd	nd	[31]
	*M. feriruminatoris*	Wild R	14/OD_0492	D	nd	un	nd	TraE detected	GenBank LR739237.1
			8756-13	D	nd	un	nd	TraE detected	GenBank LR739235.1

^1^ Small R: Small ruminant; Wild R: Wild ruminant. ^2^ D: CDSs of the MICE backbone were detected. ^3^ nd: not determined. ^4^ un: unknown. ^T^: type strain.

**Table 2:** *Mycoplasma* phages and prophages.

Phylogenetic Group	Species	Host ^1^	Strain ^2^	Phage Name	Morpho-logy ^3^	Genome (Size in Kbp)	Reference
Hominis	*M. agalactiae*	Wild R	14628	MAgV1 ^4^	nk ^6^	dsDNA (34)	[56]
	*M. alkalescens*	Cattle	ATCC 29103	MAV1-like	nk	nk	This study
	*M. arginini*	WHR	HAZ145	MAV1-like	nk	nk	This study
	*M. arthritidis*	Rodent	PG61 *	MAV1	nk	dsDNA (16)	[57,58,59]
	*M. bovigenitalium*	Cattle	51080	MAgV1-like	nk	nk	[41,56]
	*M. bovirhinis*	Cattle	nd	Br1	PH, LT	nk	[54,55]
			HAZ141_2	nd ^5^	nk	dsDNA (54)	[60,61]
	*M. bovis*	Cattle	RM16	MAgV1-like	nk	nk	This study, [62]
			3308MB	P1-like	nk	nk	This study
	*M. conjunctivae*	Small R	HRC/581 ^T^	MAgV1-like	nk	nk	[32,56]
			NCTC 10147	MFV1-like	nk	nk	This study
	*M. fermentans*	Human	PG18 ^T^*	MFV1	nk	dsDNA (16)	[63]
	*M. hominis*	Human	LBD4	MHoV1	nk	dsDNA (16)	[64]
	*M. hyorhinis*	Swine	GDL-1	Hr1	PH, ST	nk	[65]
	*M. hyosynoviae*	Swine	NPL3 *	MFV1-like	nk	nk	[66]
	*M. lipofaciens*	Avian	ATCC 35015	MAV1-like	nk	nk	This study
	*M. molare*	Canine	ATCC 27746	MAgV1-like	nk	nk	This study
	*M. mustelae*	Mink	ATCC 35214	MAgV1-like	nk	nk	This study
	*M. pulmonis*	Rodent	nd	P1	PH, ST	dsDNA (12)	[67,68,69]
	*M. spumans*	Canine	ATCC 19526	MAV1-like	nk	nk	This study

^1^ R: ruminants; WHR: Wide host range. ^2^ The asterisk indicates that similar prophage-sequences were been reported in other strains. ^3^ PH: Polyhedral head; LT: long tail; ST: short tail. ^4^ MAgV1 was initially designated as prophage 14628 [56]. ^5^ nd: not determined. ^6^ nk: not known.
